# Intrinsically motivated oculomotor exploration guided by uncertainty reduction and conditioned reinforcement in non-human primates

**DOI:** 10.1038/srep20202

**Published:** 2016-02-03

**Authors:** Nabil Daddaoua, Manuel Lopes, Jacqueline Gottlieb

**Affiliations:** 1 Department of Neuroscience, Columbia University, New York City, NY, United States; 2 The Kavli Institute for Brain Science, Columbia University, New York City, NY, United States; 3 INRIA, Bordeaux, France

## Abstract

Intelligent animals have a high degree of curiosity – the intrinsic desire to know – but the mechanisms of curiosity are poorly understood. A key open question pertains to the internal valuation systems that drive curiosity. What are the cognitive and emotional factors that motivate animals to seek information when this is not reinforced by instrumental rewards? Using a novel oculomotor paradigm, combined with reinforcement learning (RL) simulations, we show that monkeys are intrinsically motivated to search for and look at reward-predictive cues, and that their intrinsic motivation is shaped by a desire to reduce uncertainty, a desire to obtain conditioned reinforcement from positive cues, and individual variations in decision strategy and the cognitive costs of acquiring information. The results suggest that free-viewing oculomotor behavior reveals cognitive and emotional factors underlying the curiosity driven sampling of information.

The desire to predict the future is a powerful motivator in daily behavior, and has been proposed to account for the extraordinary curiosity shown by humans and other intelligent species^1^,^2^,^3^,^4^. Curiosity - the intrinsic motivation to learn - is arguably central to cognitive and social functions (ibid), but few studies have probed its mechanisms^5^,^6^,^7^,^8^. An enduring challenge in addressing this question has been to specify the internal value functions that *motivate* subjects to acquire information when this is not associated with obvious material gains^2^,^9^. While typical laboratory paradigms have focused on operant tasks where subjects seek to obtain experimenter-provided goals or rewards, it remains unclear what *internal goals* the subjects seek to achieve when they are simply curious to obtain information.

One strategy for addressing this question is based on so-called “observing paradigms”, where animals are asked to choose between two options that are equal in their physical rewards but differ in the advance information they offer. In these paradigms the subject begins a trial with uncertainty about the size of a reward, and must choose between observing one of two cues which are, respectively, predictive or not predictive about the reward. Pigeons and monkeys show a consistent preference for the informative cue, and this preference is encoded in monkey midbrain dopamine neurons and orbitofrontal cells^10^,^11^.

A key feature of observing paradigms is that they do not differentially reinforce the subjects for choosing either option, implying that a preference for informative cues derives from internal sources of motivation. However, important questions remain about the nature of this motivation.

The initial interpretation of the findings with monkeys was that the animals placed intrinsic value on resolving *uncertainty* - that is, they sought to improve the quality (certainty) of their beliefs about future outcomes^10^,^11^. A preference for the early resolution of uncertainty is consistent with economic theories^12^ and with uncertainty-based accounts of curiosity stating that individuals are motivated to close “information gaps”^1^.

However, an alternative and potentially simpler explanation, is that that the subjects merely sought to obtain *conditioned reinforcement* from a positive Pavlovian cue. According to this view, the animals’ preference in the observing task was motivated by the prospect of receiving a positive signal associated with the informative option - the cue indicating 100% large reward likelihood. This interpretation is supported by dual-models of curiosity postulating that animals are motivated both by uncertainty reduction and by “liking” for specific items^3^. In addition, it is consistent with a rich literature showing that animals automatically approach reward-predicting Pavlovian cues even when they are not rewarded for the approach actions^13^,^14^,^15^, humans and monkeys are distracted by reward-conditioned cues even when the cues are irrelevant for their task^16^,^17^,^18^, and with computational studies showing that observing-like behaviors can be simulated based only on Pavlovian drives in reinforcement learning (RL) algorithms^13^,^14^,^19^.

In sum, although the mere act of observing a reward-associated cue can be intrinsically reinforcing, it remains unclear whether this is due to conditioned reinforcement, a motivation to reduce uncertainty, or a combination of both factors.

An additional question raised by observing paradigms relates to the fact that, in natural behavior, animals sample information through active sensing behaviors - such as looking for or attending to visual cues^2^,^20^. These behaviors are faster and more fluid than the explicit decisions probed in observing paradigms, and it is unclear whether or how they express an intrinsic preference for informative cues.

A third and final question is to what extent the observing paradigms that have been used so far elicit true intrinsic motivation, given that they have used 2-alternative forced-choice tasks where the animals *had to* select an option to obtain a reward. Because the two options have equal costs in terms of effort, actions and time, it may seem unsurprising that subjects choose the informative option, and raises the question whether the animals would still choose to sample if they were allowed to opt out of the task – i.e., if sampling entailed additional costs.

In the present study we addressed these questions using a novel paradigm where monkeys used rapid eye movements (saccades) to search for or look at reward-predictive cues. To examine the full scope of the monkeys’ intrinsic motivation we placed no operant constraints on their saccade patterns – i.e., did not provide any reward incentive for a search pattern, or require the monkeys to choose between alternative cues. Finally, to tease apart the roles of uncertainty and conditioned reinforcement we manipulated the monkeys’ prior beliefs about the trial’s reward probability.

We show that the monkeys were intrinsically motivated to sample reward cues and that this motivation derived from both uncertainty reduction and conditioned reinforcement independently of the extrinsic rewards of the task. We also show that, above and beyond a shared sensitivity to these factors, individual monkeys differed in their overall willingness to sample, suggesting that there is individual variation in decision strategy and/or the cost of sampling information. The results suggest that oculomotor behavior reflects cognitive, emotional and individual factors that are dissociated from the operant rewards of a task and shape the individual’s curiosity and intrinsic motivation.

## Results

Two monkeys performed two tasks where they could freely choose whether to view reward predictive cues. In the first, ***active search task***the monkeys had the opportunity to search for a reward cue hidden in a placeholder display, while in the second, ***free-information task*** the monkeys were free to make a saccade to a visible peripheral cue. We describe the monkeys’ behavior in the two tasks, followed by RL model simulations of behavior on the active search task.

### Active search task

**Figure 1:**
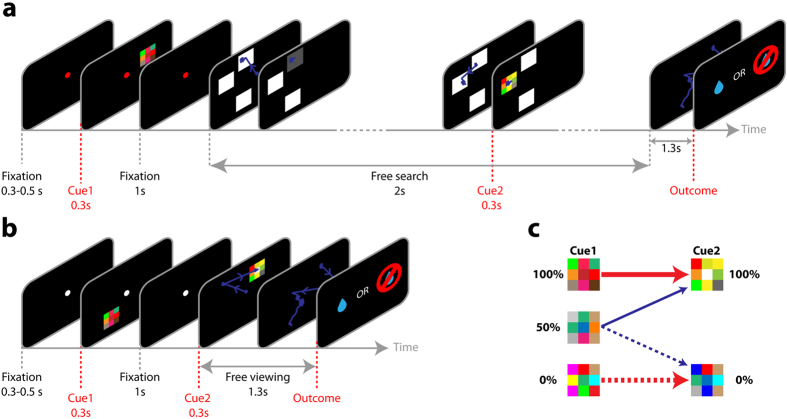
The tasks. (**a**) In the ***Active search task*** the monkey initiated each trial by maintaining gaze on a central fixation point while cue1 appeared in the periphery for 0.3 s. After an additional 1 second, the fixation point disappeared and was replaced with a display containing 3 white placeholders. The monkeys could freely examine during a 2 second “free search” period, when maintaining gaze within a 2 degree window centered on a placeholder for 300 ms caused it to reveal the underlying pattern (a gray square or an additional cue). In the example illustrated, the monkey first uncovered an uninformative gray square and later found cue 2 at the middle location. The search display then disappeared, and after an additional 1.3 s delay (blank screen), the trial ended with a tone accompanied by the outcome (a reward or a lack of reward). The timing of the reward was fixed, and its probability was entirely determined by cue 1 independently of the behavior in the search phase. (**b**) ***Free information*** task began with the same *cue 1* and fixation periods as in the active search task, was different in that, rather than presenting a placeholder display, showed a visible cue 2 pattern signaling 0% or 100% likelihood for 0.3s in the periphery. After an additional 1 s delay the trial ended with a tone and the final outcome (reward or no reward). (**c**) Transition statistics. If cue 1 signaled 100% or 0% reward likelihood, cue 2 confirmed this prediction; if cue 1 signaled a 50% reward likelihood, cue 2 was equally likely to signal a positive or a negative outcome. We equated visual statistics across all cue 1 types by ensuring that each image serving as cue 1 was followed by 2 possible images at cue 2 (each with 50% likelihood), and that cue 2 was randomly located in the visual display. Solid and dashed arrows denote transitions to, respectively, positive and negative cue 2, and red and blue colors indicate trials when cue 2 delivers redundant or new information.

In the active search task, the monkeys began each trial by achieving central fixation and viewing a first cue that provided initial information about the trial’s reward likelihood (Fig. 1a, cue 1 signaling 0%, 50% or 100% reward likelihood). After cue 1 presentation, the fixation point was removed, releasing the monkeys’ gaze, and a display consisting of 3 white placeholders appeared on the screen. One placeholder masked an additional reward cue (cue 2), and the monkeys had the opportunity to search for cue 2 by maintaining gaze on each placeholder for 300 ms to reveal the underlying pattern.

Cue 2 was hidden on all trials at a randomly selected location, and signaled a 0% or 100% reward probability – and therefore the monkeys had the opportunity to obtain perfect information by revealing this cue. However, the monkeys were under no obligation to reveal the cue. The trial’s outcome (reward or a lack of reward) arrived at a fixed delay of 3.3 seconds after fixation point offset, strictly according to the probability that had been signaled by the first cue and independently of the search pattern. Therefore, to the extent that the monkeys did decide to search for cue 2, this decision had to be intrinsically motivated.

To characterize the factors that shaped the monkeys’ motivation, we used trials in which cue 1 signaled 0%, 50% or 100% reward likelihoods (Fig. 1c). We reasoned that, if the monkeys were motivated to reduce uncertainty they should search for cue 2 *only* when the initial cue signaled 50% reward likelihood, but not when cue 1 had already removed all uncertainty (0% or 100% likelihood). By contrast, if the monkeys were motivated to view a positive, Pavlovian cue, their search should scale monotonically with the prior reward likelihood, and be maximal for a 100% cue. (Note that, while we could have in principle sampled intermediate probabilities (e.g., 25% or 75% likelihoods), any such probabilities could introduce effects related to skew – the bias toward one of the outcomes^21^ - which are best analyzed in conditions that have more than two outcomes (e.g., multiple levels of reward). Therefore, we chose to focus on the set of conditions that best distinguish between the effects of uncertainty and reward likelihood, and leave questions related to skew for future investigations.

**Figure 2:**
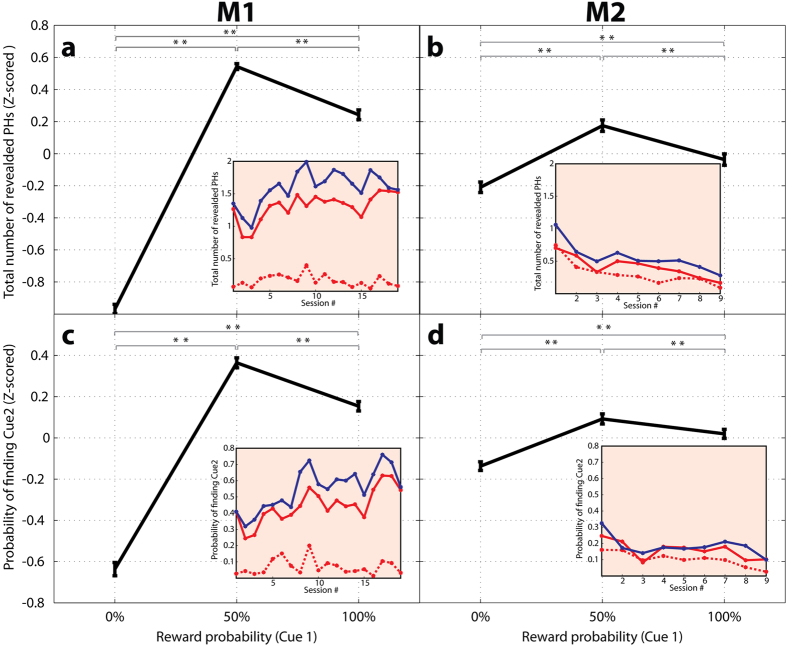
Behavior on the active search task as a function of the reward probability signaled by cue 1 In (**a**,**b**) the points show the mean and standard errors across all testing sessions, after z-scoring across cue type within individual sessions. In (**c**,**d**) we computed the probability of finding cue 2 in each session, and show the mean and standard errors of these probabilities, z-scored across all sessions. Stars indicate p < 0.025 (Wilcoxon test). The **insets** in each panel show the average of the raw data per session. The dotted red trace indicates 0% cue 1, the solid red trace shows 100% cue 1 and the solid blue trace shows 50% cue 1.

The two monkeys showed different overall levels of willingness to search for cue 2. When a reward was at stake (50% or 100% cue 1; solid blue or red traces), M1 sampled multiple placeholders and frequently discovered cue 2, and his willingness to sample increased over consecutive sessions (Fig. 2a,c, insets; logarithmic fit of the number of sampled placeholders at 50% likelihood, significant positive coefficient of 0.21, 95% confidence interval [0.08967, 0.3316], R^2^ = 0.4425). In contrast, M2 showed much less willingness to sample in initial sessions and his rate of sampling declined over time (Fig. 2b,d, insets; logarithmic coefficient of −0.2724 ([−0.3867, −0.158], R^2^ = 0.8192). This suggests that individuals vary in their intrinsic motivation to sample information, a point to which we return below.

However, despite the differences in their *overall* sampling rates, both monkeys showed a common dependence of sampling on the beliefs conferred by the initial cue (Fig. 2, main panels). Consistent with a desire to reduce uncertainty, both M1 and M2 had more vigorous search after a 50% relative to a 100% cue 1 (Fig. 2a–d, and blue vs. solid red traces in Fig. 2a–d, insets). A significant difference between 50% and 100% cues was found in the number of unmasked placeholders (Wilcoxon test on the z-scored data, M1, p < 10^−23^; M2, p = 0.0033; Fig. 2a,b) and the probability of revealing cue 2 (M1, p < 10^−5^; M2, p = 0.0288 Fig. 2c,d). This peak of the sampling function at 50% likelihood suggests that uncertainty reduction motivates information-seeking behavior above and beyond reward probability.

Surprisingly however, consistent with a desire to view a positive cue, search was also highly vigorous in trials with a 100% prior reward probability in which the monkeys had no uncertainty. Trials with a 100% relative to 0% cue 1 were associated with much higher numbers of revealed placeholders (Fig. 2a,b; M1, p < 10^−30^; M2, p < 10^−3^) and a higher probability of finding cue 2 (Fig. 2c,d; M1, p < 10^−6^; M2, p < 10^−3^). The vigorous search that the monkeys showed when they had *no prior uncertainty* but expected a positive outcome, suggests that an important component of their motivation was related to a desire to view a positive cue, even when viewing this cue brought no information and was not necessary for obtaining the reward.

As shown in the insets in Fig. 2a–d, the uncertainty and reward effects remained highly consistent across testing sessions in each individual monkey.

#### Alternative explanations

The free-viewing behavior shown by the monkeys could not be directly shaped by the operant rewards of the task, as these rewards were not contingent on any search pattern. Nevertheless, because the visual cues provided information *about* the reward, we considered several scenarios in which these rewards may have indirectly shaped the monkeys’ actions. We examined whether the search patterns could be explained by incomplete learning of the cues, or by global effects on arousal, trial by trial learning, or misunderstandings of the operant demands of the task.

#### Incomplete learning of the cues

**Figure 3:**
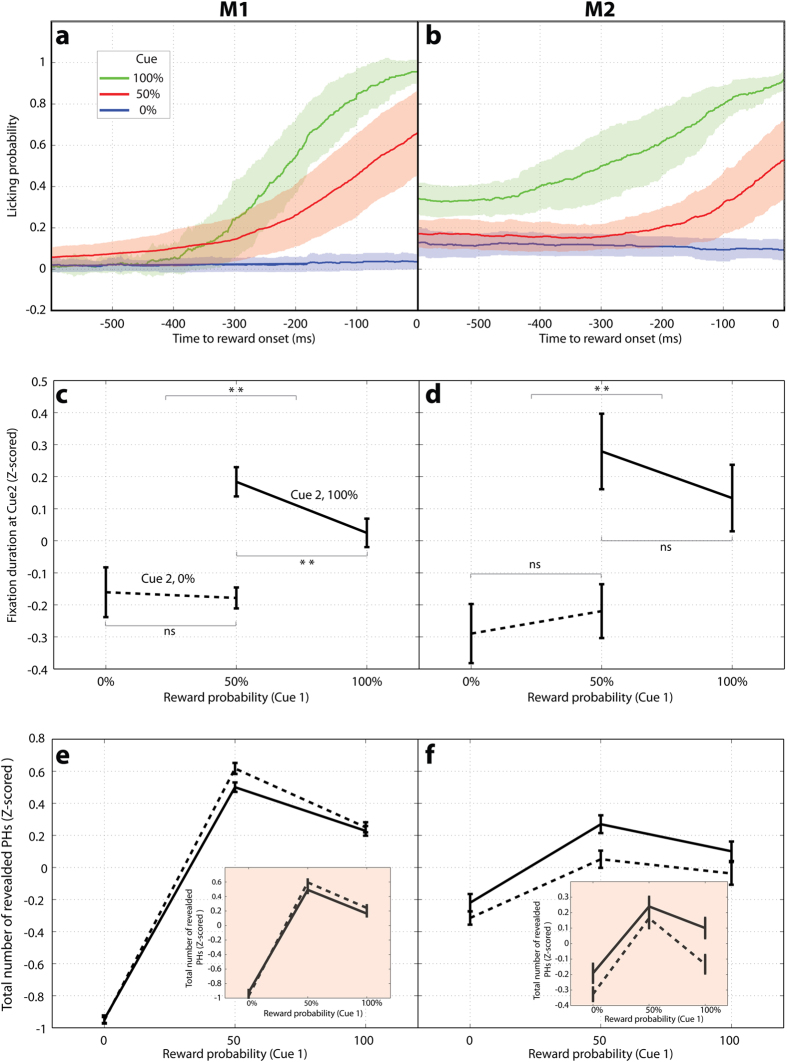
Control for learning, arousal and prior trial effects. (**a,b**) Licking behavior during the training sessions where the monkeys passively viewed cues signaling 100%, 50% or 0% probability of reward. Each trace shows the average and standard error of the licking probability for all the cues of a given type, calculated in 1 ms windows aligned on reward onset (time 0). (**c,d)** Fixation durations on cue 2 as a function of the reward signaled by this cue (solid, 100%, dotted, 0% likelihood) and the prior probability signaled by cue 1 (x-axis, 0%, 50% or 100%). Stars indicate p < 0.025 (Wilcoxon test, corrected for multiple comparisons), and *ns* indicates no significant difference (all p values were higher than 0.05).(**e,f)** Search behavior as a function of cue 1 (x–axis) for the subset of trials for which the previous trial ended in a reward (solid) or a lack of reward (dotted). The **insets** show the analogous analysis for the subset of data in which cue 2 had not been revealed in the previous trial.

One possible explanation for the robust sampling following a 100% cue is that the monkeys had residual uncertainty regarding the reliability of this cue, and sought out cue 2 as additional confirmation. Two considerations argue against this possibility. First, an explanation in terms of residual uncertainty cannot explain why sampling was much more vigorous after a 100% relative to 0% cue, as these cues had identical reliability and training histories. Second, examination of the monkeys’ anticipatory licking shows that licking was highly selective and reached constant levels during the training phase of the task - when the monkeys were learning the cues before testing on the main tasks began (Fig. 3a,b; M1, regression of lick probability as a function of session for the last 10 training sessions, p = 0.25, p = 0.39, and p = 0.93 for, respectively, 100%, 50% and 0% cues; M2, p = 0.11, p = 0.11, p = 0.25). Therefore the monkeys had acquired stable representations of the meaning of all cues before performing the active search task, speaking against an interpretation in terms of residual uncertainty.

#### Reward effects on saccade latencies

Higher reward expectation and higher uncertainty produce higher arousal, raising the question whether the saccadic behavior reflected global arousal rather than a specific desire to harvest information. In our task however, the monkeys had to maintain fixation on a placeholder for 300 ms in order to unmask the underlying pattern – a relatively long interval that required them to *slow down* their saccades in order to reveal cue 2. Moreover, when the monkeys revealed cue 2, their fixation durations were longer if the cue signaled a positive relative to a negative outcome (Fig. 3c,d; 2-way ANOVA main effect of cue 2 reward, M1, p = 0.0002; M2, p = 0) and, for monkey M1, were longer if the cue resolved prior uncertainty (2-way ANOVA, post hoc tests 100% vs 50% cue 1, M1 p = 0.01, all others, p > 0.05). Therefore conditions with high sampling were associated with *longer* inter-saccadic intervals, suggesting that they engaged a purposive search for the cue rather than global motor speeding due to higher uncertainty or reward expectation.

#### Reward history

To examine the effect of local reinforcement history we divided the data into two groups of trials for which the prior trial ended with, respectively, a reward or a lack of reward (Fig. 3e,f). Monkey M1 showed no main effect of the prior trial reward, and had a highly significant dependence on cue 1 in both trial groups (Fig. 3e, 2-way ANOVA, p = 0.95 for prior trial reward, p < 10^−97^ for cue 1 probability). Monkey M2 did show a prior trial effect, being more motivated to search after a reward rather than a no-reward outcome (Fig. 3f, 2-way ANOVA, p = 0.0003 for main effect of reward), but this influence was additive and did not affect the influence of cue 1 (p = 0.0003 for cue 1 effect, p = 0.61 for interaction). To rule out a higher order interaction between the reward and discovery of cue 2, we examined the subset of the data where the prior trial did not uncover cue 2. We obtained equivalent results (Fig. 3e,f insets; 2-way ANOVA, M1: p < 10^−97^ for cue 1, p = 0.91 for reward; M2: p = 0.0012 for cue 1, p = 0.0036 for reward), showing that the rewards or cues that the monkeys recently observed could not explain their search pattern.

#### Misunderstanding of operant demands

Because cue 2 provided information about the future reward, it is possible that the monkeys misunderstood this contingency and erroneously believed that they had to reveal this cue to gain a reward. However, examination of the monkeys’ anticipatory licking argues against this interpretation.

**Figure 4:**
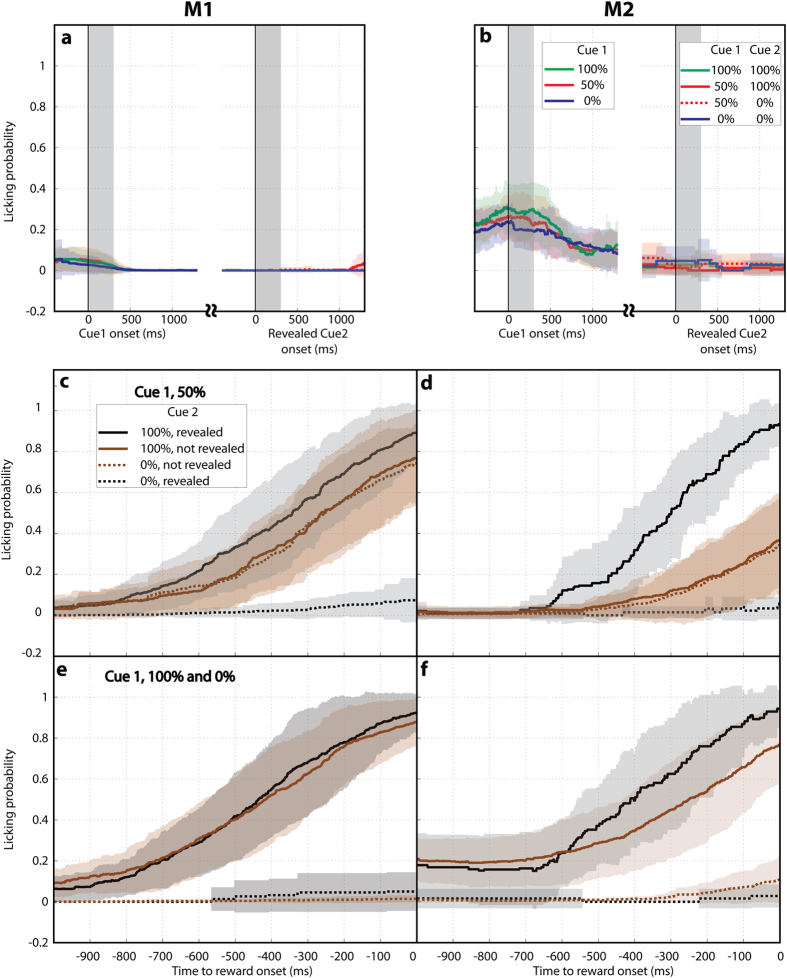
Anticipatory licking during the active search task. (**a,b**) Anticipatory licking was very low during the cue 1 and search periods, even if cue 2 was revealed. Licking probability was calculated in 1 ms windows and is shown as the average and standard deviation across sessions. Windows were aligned on the onset of cue1 and cue 2 (on trials when the latter cue was revealed), and the green shading depicts the period during which the cues were visible. The colored lines show the 3 types of cue 1 for the cue-1 aligned data, and the 4 cue1/cue 2 combinations for the cue 2 aligned data. **(c,d)** Average probability of licking aligned on the time of the outcome (time 0) on trials with a 50% cue 1 when cue 2 was revealed (black) or was not revealed (brown), and signaled a positive or a negative outcome (solid vs dotted). When cue 2 was not revealed the monkeys had an intermediate level of licking regardless of the final outcome (brown solid and dotted traces). When it was revealed, licking became high if cue 2 was positive (black solid) and dropped if it signaled a negative outcome (black dotted). (**e,f)** Average probability of licking on trials with a 100% cue 1 (solid traces) or a 0% cue 1 (dotted traces). The black and brown traces show, respectively, trials when cue 2 was or was not revealed, using the same conventions as in panels (**c,d**).

The monkeys timed their licking very precisely to the immediate pre-reward epoch – showing very low probability of licking during the cue 1 and visual search epochs (Fig. 4a,b), and starting to lick ~800 ms before the anticipated outcome, after removal of the search display (Fig. 4c–f; cf Fig. 1a). Therefore, the monkeys’ reward anticipation was not temporally linked to the cue presentation.

In trials with initial uncertainty (50% cue 1) the monkeys licked at an intermediate level if they had not revealed cue 2 (Fig. 4c,d; brown solid vs. dotted traces) but enhanced or suppressed their licking if cue 2 brought, respectively, positive or negative information (Fig. 4c,d; black solid vs. dotted traces), showing that they modulated their licking based on new information conveyed by cue 2.

However, trials in which cue 2 was redundant show that the monkeys’ interest in this cue *was not explained* by the operant use of its reward information (Fig. 4e,f). Licking on these trials was largely governed by cue 1, being highly selective and of similar vigor whether or not the monkeys discovered cue 2 (Fig. 4e,f).

The fact that the monkeys licked after a 100% cue 1 even when they had not discovered cue 2 shows that they did not consider this cue to be *necessary* to obtain the reward – i.e., they expected a reward would come based only on cue 1 (Fig. 4e,f, brown vs black traces). This conclusion is consistent with the saccadic behavior, as both monkeys had a relatively high proportion of “opt-out” trials in which they did not search at all for cue 2 even when a reward was at stake (the proportion of trials with no revealed placeholders was, after a 50% and 100% cue 1, 8.6% and 12.0% for M1, and 56.5% and 63.0% for M2).

The fact that the monkeys licked at similar levels whether or not they had discovered cue 2 suggests that they did not seek out this cue in order to improve their licking (and therefore the chance of consuming the reward). The discovery of cue 2 did not affect licking in monkey M1 (Fig. 4e solid brown vs black traces) but produced a modest enhancement in monkey M2 (Fig. 4f solid brown vs black traces; last 200 ms before the reward, p < 0.05, 1-way ANOVA). If the monkeys sought out cue 2 in order to optimize their licking, we would expect that M2 should be much more motivated to search relative to M1 – the opposite of what we observed. As a final control for this explanation, we tested M1 in a few sessions when the juice spout was placed inside of the mouth, eliminating the need for anticipatory licking. We obtained the same saccadic pattern, with significantly more placeholders revealed at 50% versus 100% likelihoods (z-scored averages, 0.6193 ± 0.0759 vs 0.3208 ± 0.0984, Wilcoxon test p = 0.0146) and at 100% vs 0% likelihoods (0.6193 ± 0.0759 vs −0.9721 ± 0.0337, p < 10–17).

In sum, these analyses rule out the possibility that the monkeys’ interest in cue 2 was explained by spurious factors including incomplete learning, arousal, recent reward history or, particularly important - a misguided attempt to use cue 2 to enhance the operant reward that they earned in the task.

### Free-information task

Whereas in the active search task cue 2 was masked in a visual display, in natural behavior stimuli can be visible at peripheral locations and be partially processed before a saccade. To examine whether reward and uncertainty also influence saccades in this scenario we used a modified version of the task where, after presenting cue 1, we showed cue 2 unmasked in the visual periphery (Fig. 1b). As in the active search task, rewards were given according to the probabilities signaled by cue 1, independently of whether the monkeys looked at cue 2.

Compared with the active search task the free-information task was easier and allowed the integration of pre- and post-saccadic information; however, it produced saccade effects of reward and uncertainty consistent with those on the active search task.

**Figure 5:**
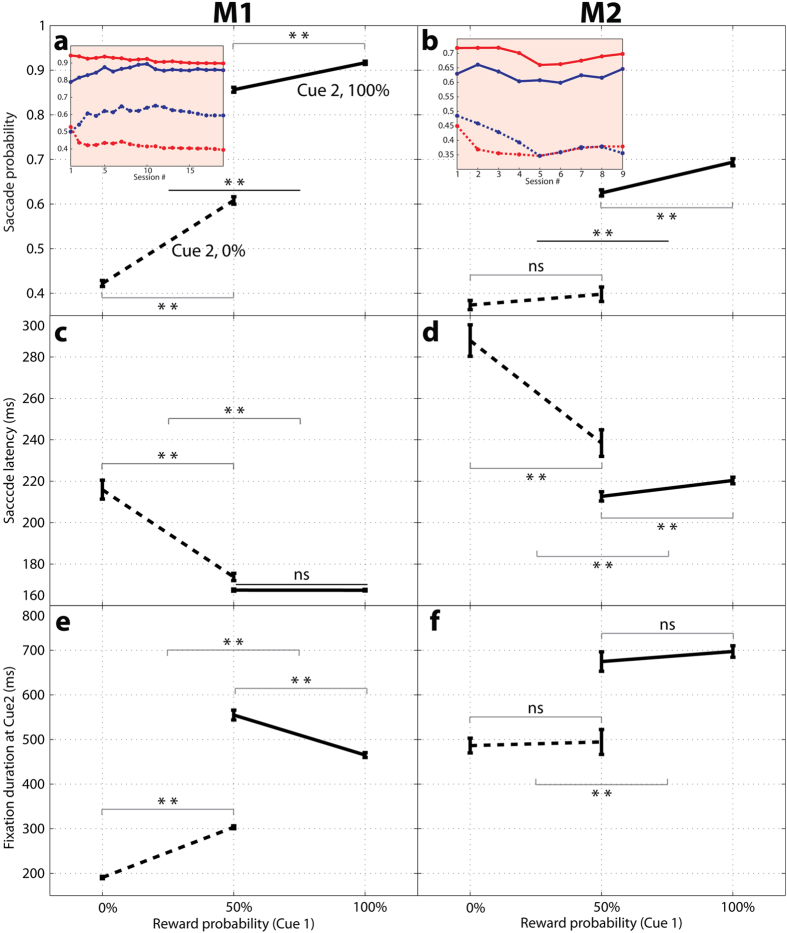
Behavior on the free information task as a function of cue 1 and cue 2. (**a,b**) The probability of making a saccade to cue 2 when this cue signaled 100% reward (solid trace) or 0% reward (dotted trace). The x-axis shows the prior probability signaled by cue 1. Each point shows the average and standard error across all sessions. The **insets** show the mean of the raw data per session. Red curves correspond to the deterministic cue 1 (solid, 100% reward, dotted, 0% reward), and the blue curves show the 50% cue 1 (solid, 100% cue 2, dotted, 0% cue 2). (**c,d**) Latency for the first saccade to cue 2 (average and standard error across all sessions), in the same format as in A,B. (**e,f**) Viewing duration (dwell times on cue 2) in the same format as in (**c,d**). In all panels, stars indicate p < 0.025 (Wilcoxon test) and *ns* indicates p > 0.025.

**Figure 6:**
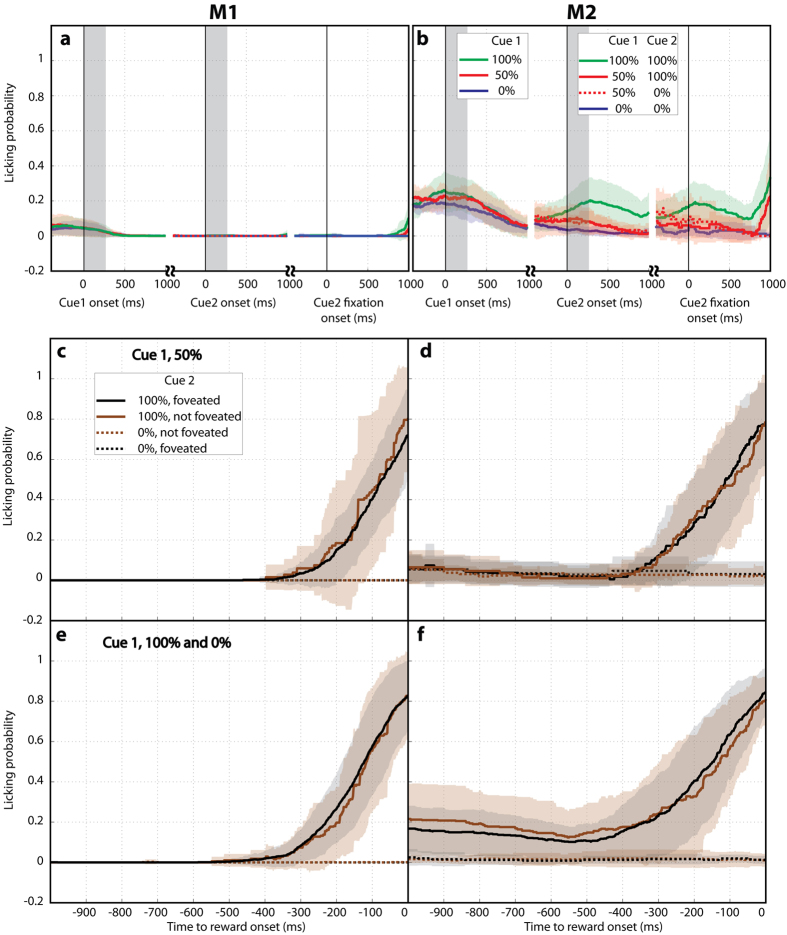
Licking behavior on the free-information task (a,b) Anticipatory licking was very low during the cue 1 and search periods, even when the monkeys made a saccade to cue 2. (**c,d**) Average probability of licking prior to the outcome delivery (time 0) on trials with a 50% cue 1. There was a significant modulation of licking by the type of outcome but not by whether cue 2 was or was not foveated. (**e,f**) Average probability of licking, aligned on the outcome delivery on trials with a 100% cue 1 (solid traces) versus 0% cue 1 (dotted traces), when the monkeys made a saccade to cue 2 (black) or did not look at this cue (brown). All conventions as in Fig. 4.

In contrast with the active search task where M2 frequently chose not to sample, in the free-information task both monkeys showed a high and stable rate of making saccades to cue 2, suggesting that this task had lower difficulty (Fig. 5a,b, insets). Linear fits of the saccade probability for 100% cue 1 as a function of session showed only a mild decrease in M1 (slope of −0.002587, 95% CI, [−0.003016, −0.002159], R^2^, 0.90) and no significant change in M2 (slope, −0.004947 [−0.01125, 0.001356], R^2^, 0.3298). Also at odds with the active search task, the monkeys’ saccade and licking behaviors show that they processed the reward information signaled by cue 2 while the cue was in their visual periphery. After a 50% cue 1, the probability of making a saccade was higher if cue 2 signaled a positive versus a negative outcome (Fig. 5a,b; M1, p < 10^−6^, M2, p < 10^−4^). Moreover, the monkeys modulated their anticipatory licking according to cue 2 even if they did not make a saccade to the cue (Fig. 6c,d).

Saccades were influenced by reward and uncertainty also in the free-information task. The effects of reward were seen in the fact that the probability of making a saccade to cue 2 was higher if this cue signaled a positive rather than a negative outcome (Fig. 5a,b, solid vs dotted traces; Wilcoxon test: M1, p < 10^−13^, M2, p < 10^−6^) and, for each type of cue 2, was higher if cue 1 had signaled a higher reward expectation (all p < 0.025). Moreover, both monkeys had significantly shorter latencies when making a saccade to a positive relative to negative cue 2 (Fig. 5c,d; Wilcoxon test, M1, p < 10^−11^, M2, p < 10^−17^), and longer post-saccadic fixation times for the positive cue (Fig. 5e,f; M1, p < 10^−50^; M2, p < 10^−31^).

The effects of uncertainty were seen in saccade timing, by comparing trials that had a positive cue 2 but a 50% or 100% cue 1 (solid traces in Fig. 5c–e). M1 had longer fixations durations after a 50% relative to a 100% cue 1 (Fig. 5e; Wilcoxon test, p = 1.02*10^−22^). M2 did not show an uncertainty effect on fixation durations, but showed shorter latencies after a 50% versus 100% cue 1 (Fig. 5d; p = 0.013). The fact that uncertainty affected post-saccadic dwell times in monkey M2 but saccade latencies in monkey M1 may reflect the monkeys’ different reliance on overt versus covert attention. M2 showed overall longer saccade latencies and lower likelihoods of making a saccade to cue 2 relative to M1 (Fig. 5b vs A and Fig. 5d vs c) suggesting that he may have more fully examined cue 2 in his visual periphery rather than during the post-saccadic fixation.

As in the active search task, there was no evidence that the monkeys misunderstood the operant demand or looked at cue 2 in order to optimize their licking responses. Similar to the search task the monkeys withheld licking during the cue-viewing epochs and began licking just before the reward (Fig. 6a,b). Second, we found that, on trials with a positive cue 2, anticipatory licking started slightly earlier if the monkeys had certain relative to uncertain prior information (100% vs. 50% cue 1; see *Methods*), confirming that they formed their reward predictions based on both cues. Finally and most important, licking was selective and of equivalent strength, whether the monkeys did or did not make a saccade to cue 2, showing that they did not consider the saccade to be necessary for obtaining the reward (Fig. 6c–f). Therefore in this task version, similar to the proactive search task, the saccadic effects of reward and uncertainty were independent of operant benefits related to receiving or consuming the reward.

### Reinforcement model

While the results presented above suggests that the monkeys were sensitive both to reward uncertainty and reward probability, these findings may have come about through several different internal value functions, perhaps combined with alternative task representations. To infer which value function was likely to motivate the saccades, we used RL models to simulate the results, focusing on the active search task that requires fewer assumptions about the integration of pre- and post-saccadic information. Rather than numerically fitting a model to the data (an exercise that is limited by the partial redundancy in the model parameterization) we focused on simulating search behavior using different internal reward functions and investigating which *type* of internal value function could reproduce the results.

**Figure 7:**
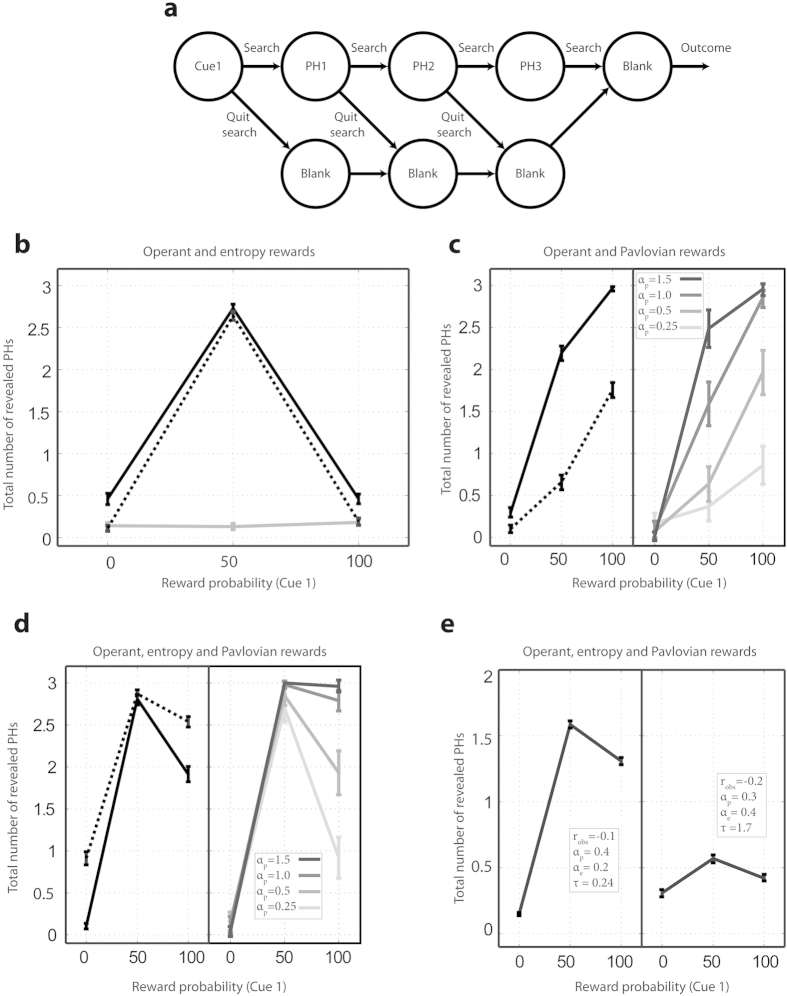
RL model simulations. (**a**) MDP used to simulate the active search task. A trial started with the first state defined by cue 1, followed by 3 decision nodes when the model could choose to search a placeholder (Ph1 – Ph3) or look at a blank screen, followed by a final blank screen and the final outcome. (**b**) Search behavior (the number of revealed placeholders) shown by a model that incorporates only an operant reward (gray solid trace) or an information reward (black traces). For the latter case, predictions are shown for models that incorporate only an information reward (solid) or an information and an operant reward (dotted). (**c**) Left panel: Search behavior shown by models that incorporate only an Pavlovian reward (solid trace) or a Pavlovian and operant reward (dotted trace). Right panel: the effect of increasing α_p_, the weight of the Pavlovian reward. (**d**) Left panel: Search behavior shown by models that incorporate all 3 reward components, when the information component depends only on the entropy of the reward distribution (solid trace), or on both the reward and visual distributions (dotted trace). Right panel: the effect of increasing α_p_, the weight of the Pavlovian reward. (**e**) Deep or shallow search curves (similar to the behaviors shown by the two monkeys) produced with different parameters of the 3-component model. In all the panels, each point shows the mean and SEM over 100 action selection iterations.

We modeled the task as a Markov Decision Process (MDP) in which the agent starts by observing cue 1, and at each step has a choice between revealing a placeholder (“Ph”) or looking at a “blank screen” (Fig. 7a). After a final “blank screen” presentation, the trial ends with a reward or a lack of reward. We simulated the precise empirical procedures that were used with the monkeys, whereby 7 different images were pre-trained as signaling 0%, 50% or 100% reward likelihoods, and were interleaved as cue 1 and cue 2 in the search task as shown in Fig. 1a,c. We used Q-learning to infer the value of inspecting a placeholder versus a blank screen and, after learning converged, simulated search behavior using a softmax function (see *Methods*).

We simulated the behavior of models driven by combinations of 3 reward types: an operant reward, an information reward and a Pavlovian reward.

The **operant reward** simulated the null hypothesis that the monkeys searched for cue 2 in order to obtain a reward, and included terms for the physical reward that the monkeys received at the final state (s_f_) and the cost of inspecting a placeholder in the current state (r_obs_). For each state (s) and action (a),


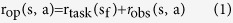


r_task_ was 1 or 0, while r_obs_ was a small negative quantity if the monkey inspected a placeholder (−1 < r_obs_ < 0) and 0 if he did not inspect.

The **information reward** simulated the possibility that the monkeys searched in order to reduce uncertainty. For each state, *s*, the information reward was equal to the negative entropy of the estimated probability distribution of the hidden future states (β(s)), analogous to the belief states used in partially observable MDPs (POMDPs):


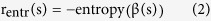


Therefore, a state that had more negative entropy (higher uncertainty) produced a higher internal reward for reducing the uncertainty.

The **Pavlovian reward** simulated the idea that the monkeys searched because they anticipated an internal reward from merely viewing a positive cue. We defined the Pavlovian reward as the square of the reward probability associated with a cue:


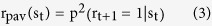


Note that, in contrast with the physical operant reward that arrives at the end of a trial (eq. 1), the Pavlovian reward depends merely on the positive associations of a given cue. This term assigns value to a state in which a cue is observed even if the operant rewards are not contingent on this observation.

Having defined these fundamental rewards, we next simulated observing behaviors based on several linear combinations of different reward types: operant rewards together with either information rewards (Fig. 7b) or Pavlovian rewards (Fig. 7b) and all 3 reward types (Fig. 7d,e).

As shown in Fig. 7b, the simulations easily reject the null hypothesis that the saccadic sampling can be explained by the operant rewards (R = r_*op*_). A model using only these rewards (solid gray trace in Fig. 7b), produces very little sampling with no dependence on the probability signaled by cue 1. This reflects the fact that in our task the operant rewards were pre-ordained and not contingent on a viewing pattern.

The black traces in Fig. 7b show the predictions based on internal reward functions that are sensitive only to information gains (solid black; R = r_ent_). Consistent with the data, these models produce sampling that is high following a 50% cue 1 but, at odds with the data, the produce sampling that is low and equivalent for 100% and 0% conditions, in which there is no reward uncertainty. Including the operant reward in the internal function (dotted trace; R = α_e_ * r_ent_ + r_op_; r_op_ = 1, α_e_ = 0.5) lowers the rates of observing because of the associated cost (r_obs_), but does not alter the shape of the function.

An important question is whether the monkeys may have sought to reduce entropy not only about the future reward but about the identity and location of cue 2 – i.e., if they were interested in simply seeing the cue. To address this question we carried out additional simulations that defined entropy over the cue locations and identities in addition to the future rewards. This new reward function led to an increase in the overall sampling (reflecting the added interest in the cue), but did not alter the shape of the function. This is consistent with the fact that the visual entropy over the cue 2 patterns was constant for all initial cues: the possible locations for cue 2 were evenly distributed across the 3 placeholders in all cases, and the conditional probability of a specific cue 2 pattern given the preceding cue 1 was equated across all trial types (i.e., each cue 1 could be followed by two possible cue 2 patterns; see *Methods*).

In sum, internal reward functions based only on information gains are not sufficient to explain the empirical search pattern, regardless of whether the monkeys’ internal task representations do or do not include curiosity about the cue patterns.

The left panel of Fig. 7c shows the predictions based on internal value functions that are sensitive only to conditioned reinforcement (solid black, R = r_*pav*_). This scenario produces sampling that is higher at 100% versus 0% rewards, but does not replicate the effect of uncertainty – i.e., produces lower sampling after a 50% relative to a 100% cue. Including a term related to the operant reward (dotted black; R = α_p_ r_pav_ + r_op_; α_p_ = 0.5) reduces sampling because of the cost of observing (r_obs_) but does not alter the shape of the function.

We considered several variants of functions based on Pavlovian rewards. First, simulations with increasing weights for the Pavlovian reward (Fig. 7c, right panel) show that, while higher weights produce a supra-linear increase for 50% cues, sampling at 50% probability never surpasses sampling after a 100% cue even when α_p_ is unrealistically large (α_p_ = 1.5, implying that monkeys value a conditioned image 50% more than they value a physical reward). Second, while in the initial simulations we squared the reward probability for conditioned reinforcement (eq. 3, implying a convex utility function), additional simulations using the non-squared probability, p, produced equivalent results. Finally, we found that including an anticipation term that simulates the delay between uncovering cue 2 and the final reward (*Methods*, eq. 8) produced equivalent results. This latter result is consistent with the task design, as the monkeys could not know in advance when they will find cue 2, and their search had to be motivated by the average temporal expectation that was fixed across the cue 1 types.

In sum, therefore, these results show that the monkeys’ sampling patterns could not be reproduced using only information or only Pavlovian rewards, under a wide range of possible task representations.

The saccade pattern could only be reproduced based on an internal function that combines all 3 types of rewards (R = r_rew_ + α_e_ r_entr_ + α_p_ r_pav_; Fig. 7d, left, solid traces). This function produces viewing behavior that is stronger after a 100% relative to a 0% cue 1 due to the Pavlovian component, and is stronger for a 50% relative to a 100% cue 1 due to the information component. Modeling uncertainty over both rewards and cue 2 locations and identities (Fig. 7d, left, dotted trace) enhances sampling but does not change the shape of the function. Increasing the relative weight of the Pavlovian reward (equivalent to reducing the weight of the operant and information rewards; right panel in Fig. 7d) changes the extent of the information modulation but maintains the shape of the function.

Finally, as shown in Fig. 7e, behaviors that resemble those of M1 and M2 can be reproduced using the 3-component model with only quantitative changes in the model parameters. The trace in the left panel of Fig. 7e shows robust search and a large modulation according to cue 1 similar to that of monkey M1 (cf Fig. 2a). The trace in the right panel of Fig. 7e shows a low and shallow search curve resembling that of monkey M2 (cf Fig. 2b). Interestingly, while the weights of the information and Pavlovian rewards were similar in the two monkeys (for M1, α_e_ = 0.2, α_p_ = 0.4 and for M2, α_e_ = 0.4, α_p_ = 0.3), the different search patterns were generated primarily by differences in the observation cost, which was double in M2 relative to M1 (r_obs_ = −0.2 vs −0.1), and in the softmax temperature parameter, which was more than 8 times higher in M2 relative to M1 (τ = 1.7 vs. 0.24). This suggests that the individual differences we found are independent of the uncertainty and Pavlovian value components, and related to individual differences in action selection strategies and the costs of gathering information.

## Discussion

While saccadic behaviors have been intensively investigated in humans and non-human primates, nearly all the investigations used instrumental tasks where saccades are motivated by extrinsic rewards^22^. In contrast, intrinsically motivated viewing behaviors have been much more difficult to characterize^2^, and computational models of such behaviors focused exclusively on sensory (bottom-up) factors^22^,^23^,^24^. Here we show, using a new observing paradigm, that monkeys are intrinsically motivated to view reward-predictive cues independently of the sensory salience or operant rewards associated with the cues. We also show that their motivation is shaped by three factors - a drive to reduce uncertainty, a desire to obtain conditioned reinforcement from a positive cue, and individual variations in decision strategy and the cost of sampling information.

A *bona fide* effect of uncertainty is suggested by the fact that the monkeys searched more vigorously for additional cues if they had a 50% rather than a 100% reward expectation. A separate, distinct effect of conditioned reinforcement was shown by the fact that the monkeys also searched vigorously in conditions in which they had no uncertainty, but had a high reward expectation. RL simulations showed that this saccadic behavior could not be reproduced by internal value functions that included *only* an information-based component, or *only* a Pavlovian component of the type that had been considered in previous investigations^19^. Instead, under a broad range of assumptions about the monkeys’ internal task representations and relative weights of the value functions, the saccadic behavior was consistent with a dual-component model of intrinsic valuation that assigned value both to uncertainty reduction and to obtaining conditioned reinforcement from positive cues^13^,^14^,^19^. Our behavioral and modeling approaches cannot, of course, exclude the possibility that the monkeys had more complex non-monotonic reward functions that could explain the results. However, given that such functions have yet to be fully characterized, the present interpretation seems the most parsimonious one.

Our finding that uncertainty reduction played a distinct role is consistent with economic theories proposing that decision makers have intrinsic preferences for the early resolution of uncertainty^12^ and with accounts of curiosity as an attempt to fill an “information gap”^1^. Both theories suggest that, even when subjects cannot increase their extrinsic gains, they value internal gains in information.

Viewed from this perspective, however, the finding that conditioned reinforcement also had an effect is puzzling, because in our paradigm viewing a positive cue did not increase the likelihood of an extrinsic reward *or* bring new information. The role of conditioned reinforcement we find is consistent with previous results showing a strong influence of Pavlovian cues^13^,^14^,^15^,^16^,^17^,^18^ and extends these results in two ways. First, our results show that Pavlovian cues not only elicit reactive behaviors but also motivate monkeys to engage in proactive search. Second, we show that this motivation persists even if the cues bring no new information. The functional benefit of this positivity-bias is not fully understood, but possible hypotheses, testable in future investigations, are that the bias is a heuristic that helps detect positive cues in complex environments, and/or that it facilitates learning by inducing positive brain states conducive to mental activity and thought^25^,^26^.

Our finding that a dual-component value function guides oculomotor behavior is consistent with more recent dual-process psychological theories suggesting that curiosity arises both from a desire to close “information gaps” (reduce uncertainty, or harvest information), and as a mere feeling of “interest” or “liking” of pleasurable items (conditioned reinforcement from positive cues)^3^. At the same time, we found that monkeys showed individual variation in their overall willingness to sample that was above and beyond these factors. This suggests that there are other, individually variable determinants of intrinsic motivation that may be related to an individual’s learning styles and the cognitive costs for assimilating information. Therefore our results suggest that oculomotor behavior is a useful model system for understanding the cognitive and emotional influences that determine curiosity in a range of conditions^2^.

## Experimental Procedures

### General methods

Data were collected from two adult male rhesus monkeys (*Macaca mulatta)*. Experiments were performed in accordance with appropriate guideline and regulations. All experimental protocol were approved by the Animal Care and Use Committees of Columbia University and New York State Psychiatric Institute as complying with the guidelines within the National Institutes of Health *Guide for the Care and Use of Laboratory Animals*. Visual stimuli were presented on a Mitsubishi Diamond Pro 2070 monitor (30.4 × 40.6 cm viewing area) located 57 cm in front of the monkey. The precise timing of stimulus presentation was measured using a diode fixed to the top left corner of the monitor to detect the onset of a refresh cycle. Eye position was recorded using a video eye tracker system (Applied Science Laboratories, model 5000) and digitized at 240 Hz. Licking was measured by means of a custom made device, using an infrared beam that was projected between the monkey’s mouth and the reward spout and produced a transistor–transistor logic pulse each time it was interrupted by protrusions of the monkey’s tongue.

### Reward cues

The cues signaling reward likelihood were 3 × 3 colored checkerboards that were equated for luminance (0.3 cd/m^2^) and size (3.0° ×3.0° on a side). The color of each constituent square in a cue was randomly drawn from a 5-color set (C1–C5) generated in the DKL color space (C1: 3R, 104G, 204B; C2: 196R, 43G, 131B; C3: 246R, 219G, 133B; C4: 110R, 233G, 170B; C5: 169R, 169G, 171B).

Each monkey was trained with a set of 7 cues, one of which signaled a probabilistic outcome (50% reward) and the remainder signaled deterministic outcomes (3 cues signaled 0% reward, and 3 signaled 100% reward). A distinct set of cues was used for each monkey, chosen so that each cue in a set differed from the others in at least 6/9 of the constituent squares.

### Training and testing

The training procedures were identical for M1 and M2. Each monkey was first familiarized with the significance of a cue in a passive viewing task, where the monkey maintained fixation on a central point, viewed a cue that was presented for 300 ms at 8° eccentricity to the left or right of fixation and, after an additional 1000 ms fixation period, received a reward with the probability assigned to the cue. The cue was no longer visible by the time of the reward and there was no consistent tendency for the monkeys to look at the cue location at the time of the reward. Therefore, the monkeys were never directly or indirectly reinforced for making a saccade to a cue.

Reward sizes were constant throughout training and testing sessions, and were 180 ms valve opening times for M1 and 250 ms for M2. Each cue was trained for several hundred trials, and learning was verified through anticipatory licking before delivery of the reward.

During the active search and free-information tasks, the position of cue 1 was randomized to fall in one of the 4 quadrants at an eccentricity that was randomly jittered between 6° and 8° (in 0.1° increments). The position of cue 2 and the placeholder display was similarly randomized within the 3 quadrants that had not been occupied by cue 1. In the active search task, the angular position of the placeholders was additionally jittered by 15° around the center of their respective quadrants, ensuring that the search display occupied slightly different positions from trial to trial and minimizing position repetition effects.

When constructing pairs of cue 1 and cue 2 we ensured that, in addition to signaling the desired reward likelihoods, the pairs had constant visual statistics. Each cue pattern that appeared as cue 1 was followed, with equal probability, by one of two possible patterns at cue 2. Thus, a cue 1 pattern signaling 50% reward was followed, with equal probability, by two possible cue 2 patterns signaling 0% or 100% likelihood. A cue 1 pattern signaling 0% or 100% reward was followed, with equal probability, by one of the other two patterns signaling the same reward likelihood. This ensured that (1) cue 2 was visually distinct from cue 1 even when it delivered redundant reward information and (2) the visual entropy of the cues (the probability of viewing a cue 2 image, given a prior cue 1) was equated across trial types.

Active search and free viewing trials were randomly interleaved in daily testing sessions. During these tasks, the monkeys’ only operant requirement was to initiate a trial by fixating a 0.5° diameter central point and maintaining gaze within 2° of this point for 1,300 ms during and after the presentation of cue 1 (Fig. 1a,b). If the monkeys broke fixation during this period, the trial was aborted without reward and immediately repeated until correctly completed, with the only difference that cue 1 appeared at a new randomly selected location. If they successfully maintained fixation through cue 1 presentation, the monkeys received rewards according to the probabilities signaled by the cues independently of their visual search or licking patterns.

### Data analysis

Saccade latencies were computed based on the eye velocity and acceleration profiles^27^. In analysis of saccade behavior on the active search task, our statistical power was limited by the low level of sampling and downward trends shown by monkey M2 (Fig. 2c,e, insets). To overcome these limitations we focused on analysis of z-scored data, which minimizes the impact of extraneous factors and focuses on the effects of the prior probability signaled by cue 1. In all other instances (session by session data for active search (insets in Fig. 2a–d), licking behaviors and the results on the free-information tasks) we used the raw values obtained from each monkey.

To examine time trends in the search behavior we fit the active search data to a logarithmic equation:


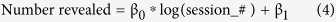


In the free information task, we fit the probability of making a saccade to cue 2 to the linear equation:


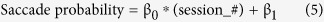


The form of these models was chosen empirically based on data patterns and is not meant to imply specific underlying mechanisms.

To ascertain that, in the free-information task the monkeys modulated their licking also based on cue 1, we compared the timing of anticipatory licking on rewarded trials in which cue 1 had delivered uncertain or deterministic information. Using the average reward-aligned licking curves (e.g., Fig. 6e,f) we compared the time at which the curve attained a criterion level of licking following 100% or a 50% cue 1 and computed the time difference between these trial types. Across all possible criteria (licking probability ranging from 0.1 to 0.7) differences were positive (M1, mean ± (SD) of 53.93 ± (4.32)ms; M2, mean ± (SD) of 62.65 ± (30.66)ms), showing that licking started earlier if the monkeys had advance reward information based on cue 1.

### RL model simulations

Using the MDP shown in Fig. 7a, we simulated an agent that seeks to maximize the cumulative discounted reward according to standard RL formula:


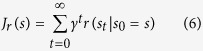


After pre-training a set of cues as signaling different reward likelihoods, we presented the model with a random mixture of cue 1 probabilities and allowed it to iterate recursively to learn action values using a Q-learning algorithm. After the Q-values converged, we simulated decisions between an observing or non-observing action using the softmax function:


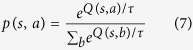


We used γ = 0.9, r_obs_ = −0.1 and τ = 0.2 unless otherwise indicated. The mean and standard errors shown in Fig. 7b–e were computed using the Q-values after convergence, by running 100 policy sampling simulations.

For completeness we also ran model simulations using a more complex Pavlovian reward that depended on temporal anticipation and γ, the temporal discount factor:


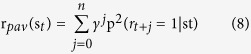


We found no changes in our predictions, and thus describe the simplest model in the main text.

In the *Results* sections we focus on the effects of varying the relative weights of the Pavlovian rewards, and of the cost of observing, r_obs_, and the temperature factor, τ. Changing the temporal discount factor will modify the weight of the operant reward, which is equivalent to changing the relative weight of the Pavlovian/information rewards.

## Additional Information

**How to cite this article**: Daddaoua, N. *et al*. Intrinsically motivated oculomotor exploration guided by uncertainty reduction and conditioned reinforcement in non-human primates. *Sci. Rep*. **6**, 20202; doi: 10.1038/srep20202 (2016).
